# Protective Effect of XinJiaCongRongTuSiZiWan on the Reproductive Toxicity of Female Rats Induced by Triptolide

**DOI:** 10.1155/2022/3642349

**Published:** 2022-06-06

**Authors:** Disi Deng, Jin Yan, Wanjing Li, Yeke Wu, Keming Wu

**Affiliations:** ^1^Department of Gynaecology, Hospital of Chengdu University of Traditional Chinese Medicine, Chengdu 610072, China; ^2^Shaanxi University of Chinese Medicine, Xianyang 712046, China; ^3^Department of Gynaecology, Fujian Maternity and Child Health Hospital College of Clinical Medicine for Obstetrics & Gynecology and Pediatrics, Fujian Medical University, Fuzhou 350001, China; ^4^Department of Stomatology, Hospital of Chengdu University of Traditional Chinese Medicine, Chengdu 610072, China

## Abstract

**Background:**

Although triptolide (TP) has been widely used for the treatment of inflammatory, autoimmune diseases, and various kinds of tumors, the long experimental and clinical applications have exhibited severe reproductive system toxicity in TP-treated animals and patients. More importantly, the underlying molecular mechanism involved in TP-induced reproductive system toxicity still needs more research.

**Methods:**

Adult female Sprague Dawley rats and human ovarian granulosa cell lines were treated with TP and then treated with XinJiaCongRongTuSiZiWan (XJCRTSZW). Histological analysis and follicle count were executed using H&E staining. Hormone (E2, AMH, FSH, LH, and INH B) concentrations, inflammation indicators (IL-1*β*, IL-6, and TNF-*α*), oxidative stress indicators (SOD, GSH-Px, and MDA), apoptosis rate, protein distribution and expression (SIRT1, AMPK, and 8-OhdG), cell viability, relative protein levels (beclin-1, LC3-II/LC3-I, p62, procaspase-3, cleaved caspase-3, p-SIRT1, SIRT1, p-AMPK*α*-1, AMPK*α*-1, Akt, and p-Akt), autophagosome were detected by ELISA, commercial biochemical detection kits, flow cytometry, immunohistochemistry, CCK-8, western blotting, and transmission electron microscope, respectively.

**Results:**

XJCRTSZW administration notably improved the TP-treated pathological symptoms, including few mature follicles in the ovary and less granular cell layer, and disordered the arrangement of the follicle, lymphocytes and plasma cells infiltration, and necrosis, shedding, and follicular cystic dilatation of the granular layer follicle cells in the ovarian stroma. Furthermore, XJCRTSZW treatment observably enhanced the TP-induced reduction of primary follicles and secondary follicles numbers and decreased the TP-induced elevation of atretic follicle numbers and the expression of AMPK, SIRT1, and 8-OhdG in GCs *in vivo*. Moreover, XJCRTSZW application significantly increased the TP-induced diminishment of E2, AMH, and LNH-B concentrations, apoptosis rate, SOD and GSH-Px concentrations, and p62 protein level; however, it declined the TP-induced augmentation of MDA level, the levels of IL-1*β*, IL-6, and TNF-*α*, autophagosome, beclin-1, LC3-II/LC3-I, cleaved-caspase-3, p-AMPK*α*-1, and p-SIRT1 protein levels both *in vivo* and *in vitro*. Besides, XJCRTSZW treatment prominently enhanced the TP-induced decrease of cell viability *in vitro*.

**Conclusion:**

XJCRTSZW can alleviate TP-induced reproductive toxicity via apoptosis, inflammation, and oxidative stress both *in vivo* and *in vitro*. Moreover, XJCRTSZW ameliorates TP-induced reproductive toxicity through AMPK/SIRT and Akt signaling axis mediated autophagy both *in vivo* and *in vitro*.

## 1. Introduction

Triptolide (TP), a diterpene trioxide, is a core component obtained from Chinese herb *Tripterygium wilfordii* Hook F [1], which has been demonstrated for its anti-inflammatory, anticancer, antioxidant, and neuroprotection and immune modulation properties [2–4]. Thus, TP has been widely used for the treatment of inflammatory, autoimmune diseases, and various kinds of tumors. However, long experimental and clinical application exhibited severe reproductive system toxicity in TP-treated animals and patients. Qian et al. [5, 6] have observed infertility in male Sprague Dawley (SD) and Wistar rats treated with 10 mg/kg *Tripterygium wilfordii* for 8 weeks. Hikim et al. [7] reported that TP severely impaired the cauda epididymal sperm ultrastructure that might be the potential antifertility action of triptolide in the male rat. Besides, TP can also induce irregular menstrual, amenorrhea, and infertility in human females [8, 9]. Therefore, it is imperative to investigate the potential molecular mechanisms of TP-induced reproductive toxicity and seek the effective drugs for the treatment of TP-induced reproductive toxicity.

The molecular mechanisms involved in the toxicity of TP might encompass oxidative stress, inflammation, apoptosis, autophagy, and other responses (cellular respiration, cell cycle, P-glycoprotein, Th17/Treg balance, cAMP/PKA signaling pathway, and heat shock proteins) [10]. Autophagy is a biological process in which the membrane encapsulates the larger molecular structures (protein, RNA, and glycogen) and the damaged or aging organelles to form autophagosomes, which are then degraded through lysosomes to maintain the cell homeostasis and renewal of organelles [11]. SH-SY5Y cells treated with TP upregulated the protein level of light chain 3II (LC3II) and LC3 puncta, which suggested that TP treatment induced cell death in neuroblastoma via autophagy pathways [12]. In addition, TP induced caspase-independent autophagic death in pancreatic cancer metastatic cell lines (S2-013, S2-VP10, and Hs766T) through the suppression of the Akt/mTOR/p70S6 K signaling axis and activation of the ERK1/2 pathway [13]. Thus, the autophagic pathway might be the ideal target for the prevention or treatment of TP-induced reproductive toxicity, and pharmacologic autophagy modulators could be promising drug candidates.

XinJiaCongRongTuSiZiWan (XJCRTSZW) is a traditional Chinese medicine (TCM) compound for invigorating the kidney, nourishing blood, and promoting blood circulation, which is created by Professor Keming Wu based on many years of clinical experience. The prescription is composed of cistanche, cuscuta, raspberry, morus, rehmannia, angelica, epimedium, Caulis Spatholobi, *Cyperus rotundus*, Fructus Leonuri, wolfberry, eupatorium, Chinese yam, and dogwood. In the prescription, cistanche, cuscuta, and raspberry are used as the emperor medicines to invigorate the kidney and essence. Epimedium, dogwood, and rehmannia are minister medicines to strengthen the power of the emperor medicines to nourish the kidney. Among them, epimedium warms the kidney and yang, and rehmannia and dogwood nourish the essence and blood of liver and kidney. The rest of the medicines are all adjuvants. Among them, angelica, Caulis Spatholobi, Fructus Leonuri, wolfberry, and eupatorium nourish blood and promote blood circulation. Morus nourishes the liver and kidneys and strengthens muscles and bones; Chinese yam nourishes qi and invigorates the spleen; and *Cyperus rotundus* is the key to regulating menstruation in gynecology, which can regulate the qi and activate the blood and make all the medicines tonic without stagnation. Moreover, our group has been demonstrating the effectiveness of XJCRTSZW on premature ovarian failure (POF) [14] and polycystic ovary syndrome (PCOS) [15]. Furthermore, our previous study has been observed that XJCRTSZW depresses the excessive activation of autophagy flux of granulosa cells (GCs) through the activation of the PI3K/AKT/mTOR signaling pathway. Therefore, we speculated that XJCRTSZW might alleviate TP-induced reproductive toxicity via autophagy pathways.

In the present study, we reported that XJCRTSZW could alleviate TP-induced reproductive toxicity via apoptosis, inflammation, and oxidative stress both *in vivo* and *in vitro*. Moreover, XJCRTSZW ameliorates TP-induced reproductive toxicity through AMPK/SIRT and Akt signaling axis mediated autophagy both *in vivo* and *in vitro*. The results of this study will provide new insights and methods for the therapy of reproductive toxicity.

## 2. Materials and Methods

### 2.1. Animal

Adult female Sprague Dawley rats (age: 7-8 weeks, weight: 200–220 g) were purchased and acclimated to standard laboratory conditions for 7 days before experiments. Rats were provided with a 12 h/12 h light-dark cycle and fed with a standard diet and water *ad libitum* at (25 ± 2)°C and 40%–60% relative humidity. All the procedures were carried out strictly based on the National Institute of Health Guide for the Care and Use of Laboratory Animals. Also, the study was ratified by the Board and Ethics Committee of Chengdu University of Traditional Chinese Medicine.

### 2.2. Cell Culture

Human ovarian granulosa cell lines (cat. no. CP-H192) were purchased from Procell (Wuhan, China). Cells were maintained in complete medium for human ovarian granulosa cells (CM-H192, Procell) at 37°C with 5% carbon dioxide (CO_2_).

### 2.3. Experimental Groups and Drug Administration

For *in vivo* experiments, 25 rats with normal oestrous cyclicity (Figure S1) were randomly divided into five groups (*n* = 5), including control, TP, TP + XJCRTSZW-high, TP + XJCRTSZW-middle, and TP + XJCRTSZW-low. Rats in TP, TP + XJCRTSZW-high, TP + XJCRTSZW-middle, and TP + XJCRTSZW-low group were intragastrically administered with 400 *μ*g/kg·d TP (dissolved in saline containing 5% DMSO) for continuous 40 days to induce the reproductive toxicity, while rats in the control group were intragastrically administered with equal amount of saline including 5% DMSO. Then, rats in TP + XJCRTSZW-high, TP + XJCRTSZW-middle, and TP + XJCRTSZW-low group were intragastrically administered 24.15 g/(kg.d), 12.075 g/(kg.d), and 6.0375 g/(kg.d) XJCRTSZW (the detailed active ingredients are in Figure S2 and Table S1), respectively, whereas rats in the control and TP groups were intragastrically administered with 1 ml/100 g saline once a day, for 2 weeks. The total amount of crude drug of XJCRTSZW per dose is 120 g, which is made into an extract by the Department of Pharmacy of Chengdu University of Traditional Chinese Medicine. The concentration of XJCRTSZW in the high, middle, and low groups was determined according to the adult clinical dose of 6 kg per kilogram of body weight and the ratio of human to rat being 1 : 20 for conversion. After the rats were intraperitoneally anesthetized with sodium pentobarbital (40 mg/kg), blood was taken from the abdominal aorta. Serum was isolated and stored at −80°C for further assays. Ovary tissues and GCs were fleetly removed for subsequent analysis.

For *in vitro* experiments, medicated serum was first prepared as per the following description. 15 rats were randomly divided into three groups (*n* = 5), including control, XJCRTSZW, and coenzyme Q10 groups. Rats in XJCRTSZW and coenzyme Q10 group were intragastrically administered with 24.15 g/(kg.d) XJCRTSZW and 15 mg/(kg.d) coenzyme Q10, respectively, while rats in the control group were intragastrically administered with 1 ml/100 g saline 2 times a day for 5 consecutive days. Then, blood was taken from the abdominal aorta after the rats were intraperitoneally anesthetized with sodium pentobarbital (40 mg/kg). Serum was isolated and inactivated the complement for *in vitro* experiments. Human ovarian granulosa cell lines were inoculated into six-well plates and divided into nine groups including control, DMSO, TP, TP + coenzyme Q10, TP + XJCRTSZW, TP + XJCRTSZW + CQ, TP + XJCRTSZW + NAC, TP + NAC, and TP + CQ groups. Human ovarian granulosa cells in the TP group were treated with 200 *μ*L 100 nM TP for 12 h and then treated with 200 *μ*L medicated serum obtained from the control group as above described. Cells in the TP + coenzyme Q10 group were treated with 200 *μ*L 100 nM TP for 12 h and then treated with 200 *μ*L medicated serum obtained from the coenzyme Q10 group as above described. Cells in the TP + XJCRTSZW group were treated with 200 *μ*L 100 nM TP for 12 h and then treated with 200 *μ*L medicated serum obtained from the XJCRTSZW group as above described. Cells in the TP + XJCRTSZW + CQ group were treated with 200 *μ*L 100 nM TP for 12 h and then treated with 200 *μ*L medicated serum obtained from the XJCRTSZW group and 100 *μ*M chloroquine (CQ), respectively. Cells in the TP + XJCRTSZW + NAC group were treated with 200 *μ*L 100 nM TP for 12 h and then treated with 200 *μ*L medicated serum obtained from the XJCRTSZW group and 10 *μ*M N-acetylcysteine (NAC), respectively. Cells in the TP + NAC group were treated with 200 *μ*L 100 nM TP for 12 h and then treated with 10 *μ*M NAC. Cells in the TP + CQ group were treated with 200 *μ*L 100 nM TP for 12 h and then treated with 100 *μ*M chloroquine. Cells in the control and DMSO groups were treated with the same amount of saline and dimethyl sulfoxide (DMSO), respectively, and then treated with 200 *μ*L medicated serum obtained from the control group as above described. Subsequently, cells were maintained at 37°C with 5% carbon dioxide (CO_2_) for further 48 h.

### 2.4. Cell Counting Kit-8 Assay

Human ovarian granulosa cells were inoculated in 96-well plates with a density of 1 × 10^5^/well and cultured for 24 h at 37°C in 5% CO_2_. Subsequently, the cell count kit-8 (Dojindo Laboratories, Kumamoto, Japan) was used to detect the proliferation of cells according to the operating manual. The absorbance was recorded at 450 nm by a microplate reader (Thermo Fisher Scientific, Waltham, MA, USA).

### 2.5. Histological Assays and Follicle Count

The ovarian tissue was separated, fixed, embedded, and cut into sections. 5 *μ*m sections were stained with hematoxylin and eosin (H&E). Pictures were obtained under a microscope (DMI1, LEICA, Germany). Then, the number of primary follicles, secondary follicles, and atretic follicles was measured.

### 2.6. Enzyme-Linked Immunosorbent Assay (ELISA)

The levels of estradiol (E2), anti-Mullerian hormone (AMH), follicle stimulating hormone (FSH), luteinizing hormone (LH), inhibin B (INH B) were detected using the Estradiol ELISA Kit (PE223, Beyotime, Shanghai, China), rat anti-Mullerian hormone (AMH) ELISA KIT (YB-AMH –Ra, Ybscience, Shanghai, China), rat follicle stimulating hormone (FSH) ELISA KIT (XY-FSH-Ra, Ybscience), rat luteinizing hormone (LH) ELISA KIT (XY-LH-Ra, Ybscience), and rat Inhibin B ELISA KIT (YS-H5787, Yansheng Biology, Shanghai, China) according to the manufacturer's instructions. The serum or supernatant levels of IL-1*β*, IL-6, and TNF-*α* were also determined by rat IL-1*β* ELISA kit (ZC-36391), rat IL-6 ELISA kit (ZC-36404), and rat TNF-*α* ELISA kit (ZC-37624) (all in Zhuocai Biological Technology, Shanghai, China) based on the manufacturer's instructions. The absorbance of wells was determined with a microplate reader (Thermo Fisher Scientific) at 450 nm wavelength to analyze the sample concentration.

### 2.7. Biochemical Detection

The levels of malondialdehyde (MDA), superoxide dismutase (SOD), and glutathione peroxidase (GSH-Px) were detected using the lipid peroxidation MDA assay kit (S0131 M, Beyotime), total superoxide dismutase assay Kit with NBT (S0109, Beyotime), and total glutathione peroxidase assay kit with NADPH (S0058, Beyotime) according to the manufacturer's instructions. The absorbance of wells was determined with a microplate reader (Thermo Fisher Scientific) at 532 nm (MDA), 560 nm (SOD), and 340 nm (GSH-Px) wavelengths to analyze the sample concentration.

### 2.8. Flow Cytometric Assay

Apoptosis of GCs was evaluated by a flow cytometric assay. In brief, GCs were collected and stained with Annexin V-APC and PI (Sigma Aldrich, St. Louis, MO, USA) at room temperature for 20 min in the dark. The fluorescence of the cells was measured by flow cytometry (BD FACSVerse, Waltham, MA, USA).

### 2.9. Immunohistochemistry (IHC)

GCs separated from the ovary were immobilized with 4% paraformaldehyde for 6 h at room temperature. After being dehydrated, embedded, and cut, sections (5 *μ*m) were obtained for the IHC experiment. Sections were stained with rabbit polyclonal SIRT1 antibody (1 : 100, bs-5973R, Bioss, Beijing, China), rat monoclonal AMPK antibodies (1 : 100, 66536-1-Ig, Bioss), and rabbit polyclonal 8-OHdG antibody (1 : 100, bs-1278R, Bioss) overnight at 4°C. Subsequently, the sections were incubated with goat anti-rabbit IgG (H + L)-biotin (1 : 10000, SP-9001, Zsbio, Beijing, China) at 4°C for 30 min. The results were analyzed with the digital trinocular camera microscope (BA400Digital, McAudi Industry Group Co., Ltd.) and image analysis software Image-Pro Plus 6.0 (Media Cybernetics, USA).

### 2.10. Transmission Electron Microscopy

Ovary tissues and GCs were fixed in 3% glutaraldehyde and 1% osmium tetroxide and cut on an ultramicrotome. Then, sections were stained with 1% uranyl acetate and 0.5% lead citrate successively. The results were observed using a JEM-1400PLUS transmission electron microscope.

### 2.11. Western Blot Assay

Protein samples from GCs separated from ovary or human ovarian granulosa cells were extracted using a total protein extraction kit (BC3711, Solarbio, Beijing, China). Then, the protein concentration was detected by a protein assay kit (Beyotime). Next, protein samples were separated by 10% SDS-PAGE gel and electrically transferred to PVDF membranes (Millipore, MA, USA). After being blocked with 3% bovine serum albumin (BSA) for 1 h at room temperature, the membranes were incubated with the primary antibodies at 4°C overnight. After washing with TBST for 3 × 5 min, the membranes were incubated with goat anti-Rabbit IgG (H + L)-HRP (1 : 10000, ab6721, Abcam, Cambridge, UK) for 1 h at room temperature. Protein bands were analyzed by an electrochemiluminescence (ECL) chemiluminescence kit (WBULS0500; EMD Millipore), and the band intensity was quantified with Image-Pro Plus 6.0 software. The primary antibodies used were as follows: rabbit anti-beclin-1 (ab210498; 1 : 1,000; Abcam), rabbit anti-LC3II/I (ab128025; 1 : 1,000), rabbit anti-p62 (ab56416; 1 : 1,000), rabbit monoclonal (E61) to procaspase-3 (ab32150; 1 : 1,000), rabbit anticleaved caspase-3 (ab2302; 1 : 1,000), rabbit monoclonal (EPR2849Y) to SIRT1 (phospho S47) (ab76039; 1 : 2,000), rabbit monoclonal (EPR18239) to SIRT1 (ab189494; 1 : 1,000), rabbit anti-AMPK*α* (ab3759; 1 : 2,000), rabbit anti-p-AMPK*α* (ab194920; 1 : 2,000), rabbit polyclonal to pan-AKT (ab8805; 1 : 500), rabbit polyclonal to AKT (phospho T308) (ab38449; 1 : 1,000), and rabbit anti-*β*-actin (ab8227; 1 : 1,000).

### 2.12. Statistical Analysis

Data were presented as the means ± standard deviation. Differences among multiple groups were analyzed using one-way analysis of variance and Duncan's test using the SPSS 20.0 package (SPSS Inc. Chicago, IL, USA). The differences were considered as statistically nonsignificant and significant when *p* > 0.05 and *p* < 0.05, respectively.

## 3. Results

### 3.1. XJCRTSZW Alleviates TP-Induced Reproductive Toxicity *In Vivo*

**Figure 1:**
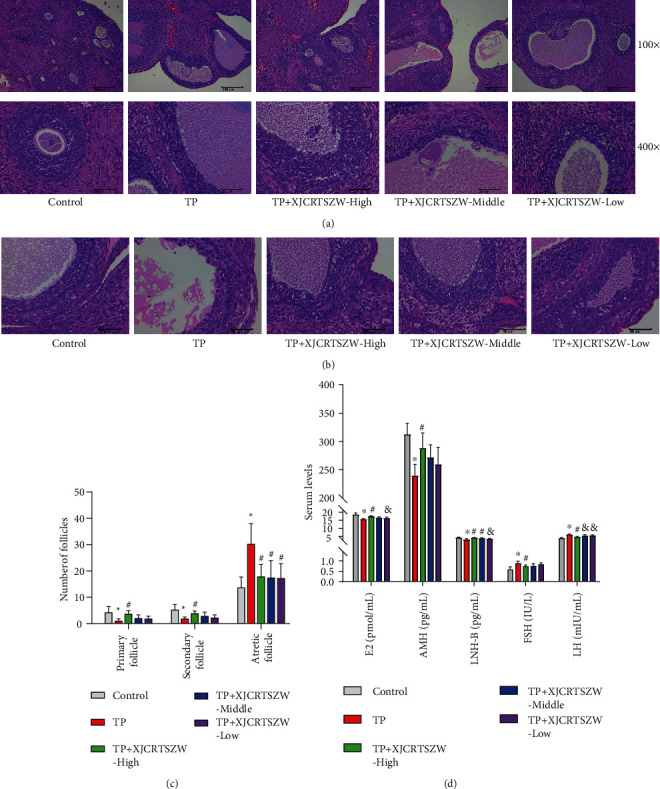
XJCRTSZW ameliorates TP-induced reproductive toxicity. (a) Histological analysis of the ovary was determined by H&E stain. (b) The number of primary follicles, secondary follicles, and atretic follicles was measured after ovary was stained with H&E. (c) The serum level of E2, AMH, LNH-B, FSH, and LH was detected using commercial ELISA kits. (d) The means ± SD of five independent samples were shown. ^*∗*^*p* < 0.05 compared to the control group. ^#^*p* < 0.05 compared to the TP group . ^&^p < 0.05 compared to the TP + XJCRTSZW-high group.

H&E staining analysis showed that there were few mature follicles in the ovary, less granular cell layer and disordered arrangement of the follicle, lymphocytes and plasma cells infiltration, and necrosis, shedding, and follicular cystic dilatation of the granular layer follicle cells in the ovarian stroma in TP-treated rats, which was observably ameliorated with XJCRTSZW treatment (Figure 1(a)). Moreover, follicle count analysis revealed that the number of primary follicles and secondary follicles was notably decreased, while atretic follicle numbers were significantly increased with TP treatment (Figures 1(b) and 1(c)). However, a high-dose of XJCRTSZW treatment prominently reversed the change of number of primary follicles, secondary follicles, and atretic follicles induced by TP treatment (Figures 1(b) and 1(c)). In addition, a high-dose of XJCRTSZW treatment markedly enhanced the TP-induced reduction of the serum level of E2, AMH, and LNH-B, whereas it declined the TP-induced elevation of the serum level of FSH and LH (Figure 1(d)). Altogether, these results suggested that XJCRTSZW relieved TP-induced reproductive toxicity.

### 3.2. XJCRTSZW Ameliorates TP-Induced Reproductive Toxicity via Apoptosis, Inflammation, and Oxidative Stress *In Vivo*

**Figure 2:**
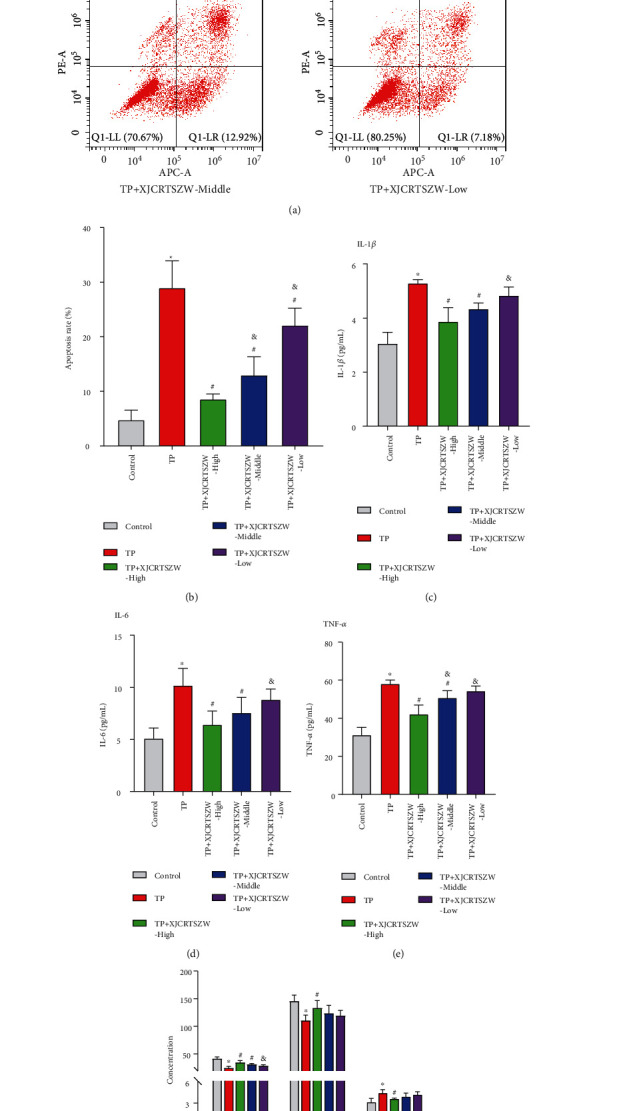
XJCRTSZW relieves TP-induced reproductive toxicity via apoptosis, inflammation, and oxidative stress. (a, b) The apoptosis rate was determined using a flow cytometry assay. (c–e) The serum levels of IL-1*β* (c), IL-6 (d), and TNF-*α* (e) were measured by ELISA. (f) The level of SOD, GSH-Px, and MDA in GCs was detected using commercial kits. The means ± SD of five independent samples were shown. ^*∗*^*p* < 0.05 compared to the control group. ^#^*p* < 0.05 compared to the TP group. ^&^Compared to the TP + XJCRTSZW-high group.

Flow cytometry analysis exhibited that the apoptosis rate was significantly enhanced in the TP group compared to that in the control group, while all the high, middle, and low-dose XJCRTSZW treatment prominently reduced the TP-induced increase of apoptosis rate. Moreover, the reduced apoptosis rate showed a significant statistical difference among the high, middle, and low-dose XJCRTSZW treatment groups (Figures 2(a) and 2(b)). Besides, both high and middle-does XJCRTSZW treatment notably declined the TP-enhanced the serum levels of IL-1*β*, IL-6, and TNF-*α*, while low-does XJCRTSZW treatment just decreased the TP-increased the serum levels of IL-1*β*, IL-6, and TNF-*α* with no statistical difference (Figures 2(c)–2(e)). In addition, TP treatment notably reduced the level of SOD and GSH-Px in GCs, which was prominently rescued with high-dose XJCRTSZW treatment. On the contrary, high-dose XJCRTSZW treatment significantly decreased the TP-induced enhancement of MDA level (Figure 2(f)). Therefore, these data indicated that XJCRTSZW relieves TP-induced reproductive toxicity via apoptosis, inflammation, and oxidative stress.

### 3.3. XJCRTSZW Relieves TP-Induced Reproductive Toxicity via Autophagy *In Vivo*

**Figure 3:**
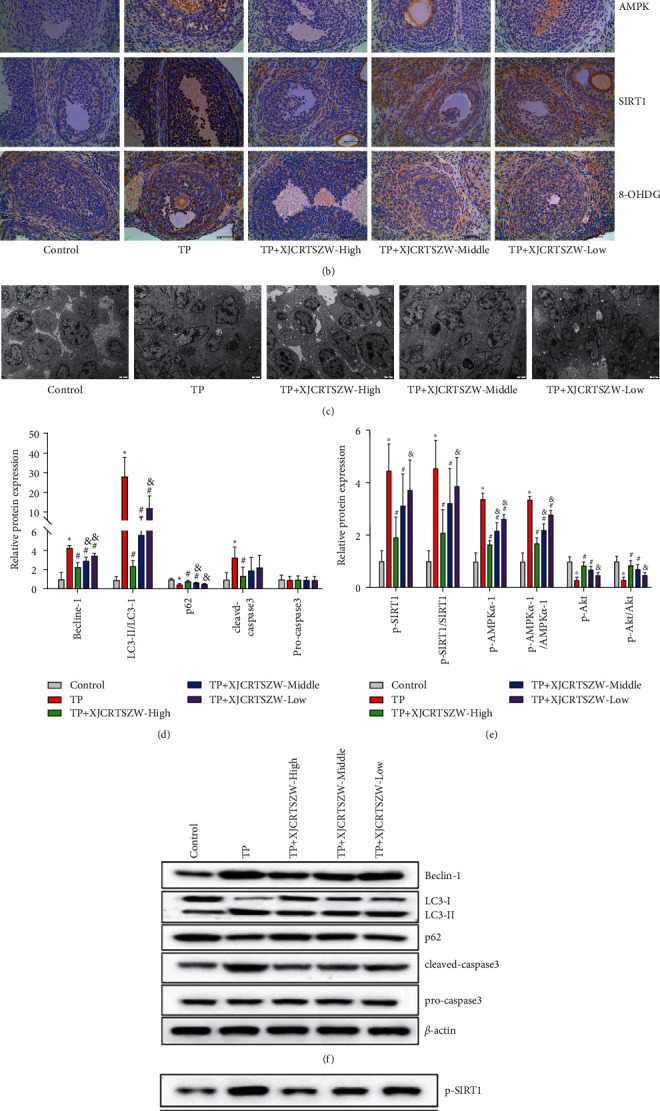
XJCRTSZW ameliorates TP-induced reproductive toxicity via autophagy by inhibiting the AMPK*α*-1/SIRT1/Akt signaling axis. (a, b) The expression level of AMPK, SIRT1, and 8-OhdG in GCs was detected using IHC. (c) The autophagosome in GCs was evaluated using a transmission electron microscope. (d–g) The protein levels of beclin-1, LC3-II/LC3-I, cleaved-caspase-3, p62, procaspase-3, p-AMPK*α*-1, p-SIRT1, p-Akt, AMPK*α*-1, SIRT1, and Akt were determined using the Western blot. The data were expressed after being normalized to *β*-actin. The means ± SD of five independent samples were shown. ^*∗*^*p* < 0.05 compared to the control group. ^#^*p* < 0.05 compared to the TP group. ^&^*p* < 0.05 compared to the TP + XJCRTSZW-high group.

IHC results showed that the expression level of AMPK, SIRT1, and 8-OhdG in GCs was notably upregulated, which was observably inverted with both high and middle-dose XJCRTSZW treatment (Figures 3(a) and 3(b)). In addition, XJCRTSZW treatment significantly reduced the TP-induced enhancement of autophagosome (Figure 3(c)). Furthermore, as shown in Figures 3(d) and 3(e), a high-dose XJCRTSZW treatment significantly reduced the TP-induced increase of beclin-1, LC3-II/LC3-I, and cleaved-caspase-3 protein levels, while prominently enhanced the TP-induced decrease of p62 protein level. Also, no statistical change was observed in the protein level of procaspase-3. Moreover, the phosphorylated protein level of AMPK*α*-1, SIRT1, and Akt was markedly elevated with TP treatment, which was notably antagonized with all high, middle, and low-dose XJCRTSZW treatments. Similarly, no statistical change was measured in the protein level of AMPK*α*-1, SIRT1, and Akt (Figures 3(f) and 3(g)). Taken together, we concluded that XJCRTSZW relieved TP-induced reproductive toxicity via autophagy by inhibiting the AMPK*α*-1/SIRT1/Akt signaling axis.

### 3.4. XJCRTSZW Enhances the TP-Induced Decrease of Human Ovarian Granulosa Cell Line Viability

**Figure 4:**
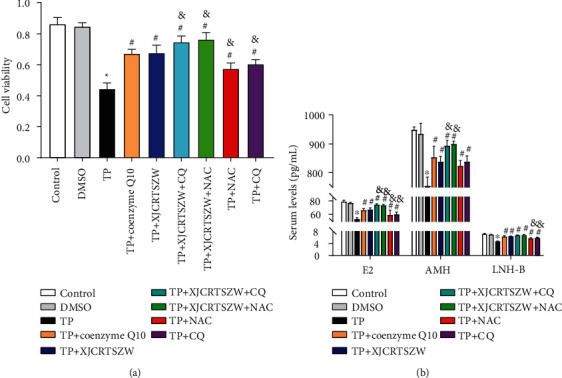
XJCRTSZW increases the TP-induced reduction of human ovarian granulosa cell line viability. (a) Cell viability was detected using CCK-8. (b) The level of E2, AMH, and LNH-B levels in the supernatant of cultured human ovarian granulosa cells was determined using commercial ELISA kits. The means ± SD of three independent samples were shown. ^*∗*^*p* < 0.05 compared to the control group. ^#^*p* < 0.05 compared to the TP group. ^&^*p* < 0.05 compared to the TP + XJCRTSZW group.

The cell viability of human ovarian granulosa cells was notably declined with TP treatment, which was prominently rescued with coenzyme Q10 (western medicine control) or XJCRTSZW treatment. Moreover, the use of CQ (inhibitor of autophagy) and NAC (inhibitor of oxidative stress) further significantly elevated human ovarian granulosa cells viability on the basis of the XJCRTSZW treatment, while the effect of CQ or NAC treatment alone was observably worse than that of coenzyme Q10 or XJCRTSZW treatment alone (Figure 4(a)). Similarly, coenzyme Q10 or XJCRTSZW treatment prominently enhanced the TP-induced reduction of E2, AMH, and LNH-B levels in the supernatant of cultured human ovarian granulosa cells. XJCRTSZW treatment combined with CQ or NAC treatment further markedly elevated the TP-induced decrease of E2, AMH, and LNH-B levels in the supernatant of cultured human ovarian granulosa cells (Figure 4(b)). Thus, we demonstrated that XJCRTSZW enhances the TP-induced decrease of human ovarian granulosa cell lines viability.

### 3.5. XJCRTSZW Ameliorates TP-Induced Apoptosis, Inflammation, and Oxidative Stress of Human Ovarian Granulosa Cells

**Figure 5:**
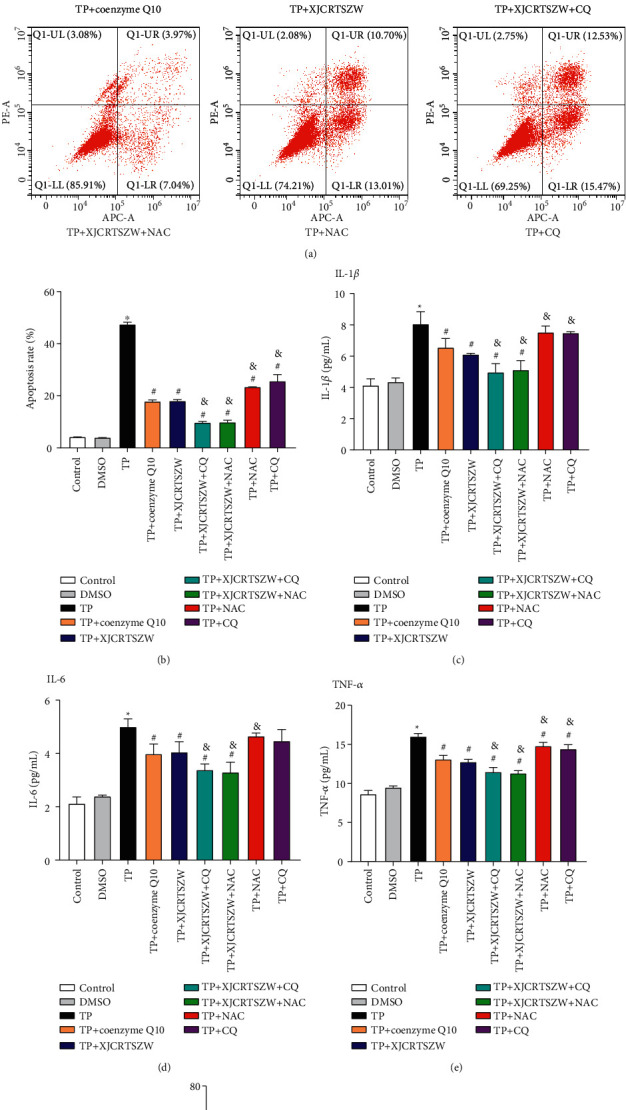
XJCRTSZW ameliorates TP-induced apoptosis, inflammation, and oxidative stress of human ovarian granulosa cells. (a, b) The apoptosis rate was determined using a flow cytometry assay. (c–e) The supernatant levels of IL-1*β* (c), IL-6 (d), and TNF-*α* (e) were measured by ELISA. (f) The level of SOD, GSH-Px, and MDA in GCs was detected using commercial kits. The means ± SD of three independent samples were shown. ^*∗*^*p* < 0.05 compared to the control group. ^#^*p* < 0.05 compared to the TP group. ^&^*p* < 0.05 compared to the TP + XJCRTSZW group.

The apoptosis rate of human ovarian granulosa cells was significantly enhanced in the TP group compared to that in the control group, while coenzyme Q10 or XJCRTSZW treatment notably declined the TP-induced increase of apoptosis rate, and XJCRTSZW treatment combined with CQ or NAC treatment further prominently decreased the TP-induced elevation of apoptosis rate (Figure 5(a) and 5(b)). The same tendency was also observed in the supernatant level of IL-1*β*, IL-6, and TNF-*α* (Figures 5(c)–5(e)). In addition, coenzyme Q10 or XJCRTSZW treatment observably increased the TP-induced reduction of SOD and GSH-Px levels in the supernatant of cultured human ovarian granulosa cells, while markedly declined the TP-induced enhancement of MDA level in supernatant of cultured human ovarian granulosa cells. Moreover, XJCRTSZW treatment combined with CQ or NAC treatment further dramatically increased the SOD and GSH-Px level, whereas it reduced MDA level in supernatant of cultured human ovarian granulosa cells (Figure 5(f)). In brief, these data suggested that XJCRTSZW ameliorates TP-induced apoptosis, inflammation, and oxidative stress of human ovarian granulosa cells.

### 3.6. XJCRTSZW Ameliorates TP-Induced Autophagy of Human Ovarian Granulosa Cells

**Figure 6:**
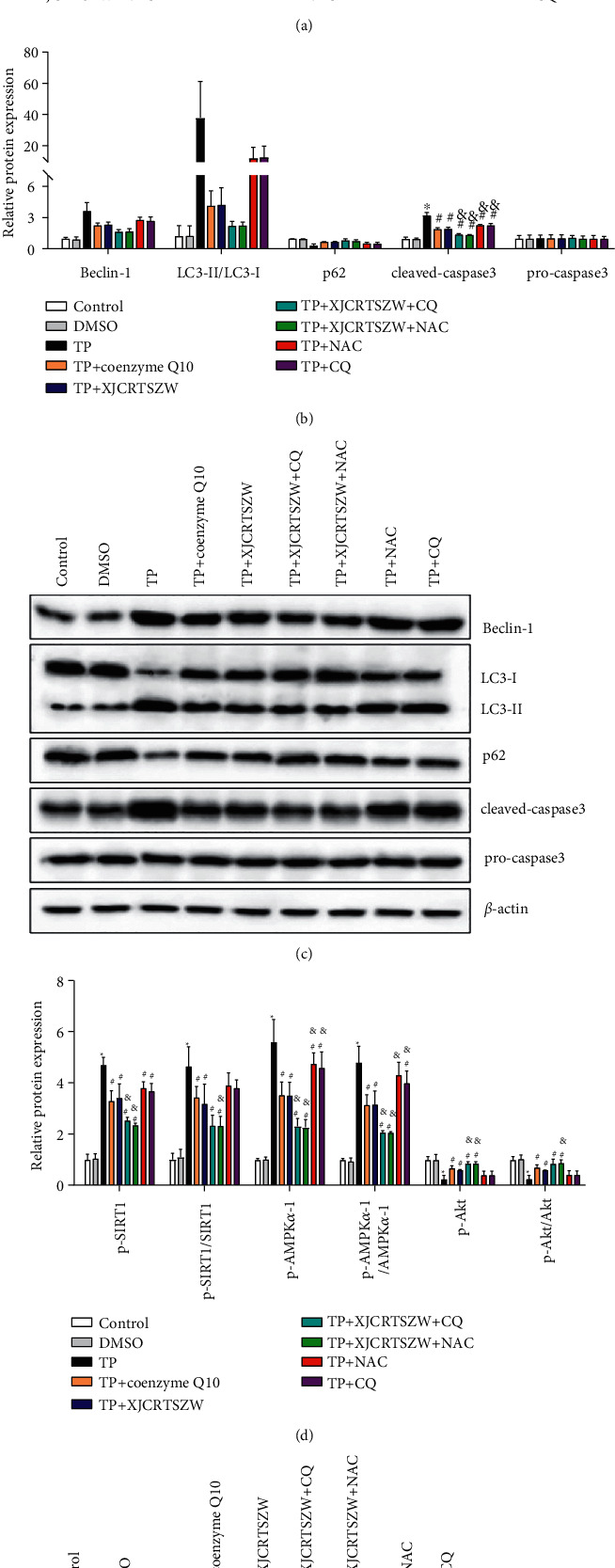
TP-induced autophagy of human ovarian granulosa cells through the AMPK*α*-1/SIRT1/Akt signaling axis. (a) The autophagosome in human ovarian granulosa cells was evaluated using transmission electron microscope. (b, c) The protein level of beclin-1, LC3-II/LC3-I, cleaved-caspase-3, p62, procaspase-3, p-AMPK*α*-1, p-SIRT1, p-Akt, AMPK*α*-1, SIRT1, and Akt was determined using the Western blot. The data were expressed after being normalized to *β*-actin. The means ± SD of three independent samples were shown. ^*∗*^*p* < 0.05 compared to the control group. ^#^*p* < 0.05 compared to the TP group. ^&^*p* < 0.05 compared to the TP + XJCRTSZW group.

Human ovarian granulosa cells treated with TP showed cell necrosis, fragmented nuclei, chromatin aggregation, as well as the disordered cytoplasmic content, the blurred structure, pyknotic mitochondria, and a lot of autophagosomes. However, coenzyme Q10 or XJCRTSZW treatment notably ameliorated these symptoms, and XJCRTSZW treatment combined with CQ or NAC treatment further prominently improved these symptoms of cultured human ovarian granulosa cells (Figure 6(a)). In addition, coenzyme Q10 or XJCRTSZW treatment markedly decreased the TP-induced enhancement of beclin-1, LC3-II/LC3-I, cleaved-caspase-3, p-AMPK*α*-1, p-SIRT1, and p-Akt protein levels, whereas it observably elevated the TP-induced decrease of p62 protein levels. Similarly, XJCRTSZW treatment combined with CQ or NAC treatment further notably reduced the protein level of beclin-1, LC3-II/LC3-I, and cleaved-caspase-3, whereas it increased the protein level of p62. No statistical change was discovered in the protein level of procaspase-3, AMPK*α*-1, SIRT1, and Akt (Figures 6(b)–6(e)). Therefore, we concluded that XJCRTSZW ameliorates TP-induced autophagy of human ovarian granulosa cells through the AMPK*α*-1/SIRT1/Akt signaling axis.

## 4. Discussion

The main results of the present study are that XJCRTSZW can alleviate TP-induced reproductive toxicity via apoptosis, inflammation, and oxidative stress both *in vivo* and *in vitro*. Moreover, XJCRTSZW ameliorates TP-induced reproductive toxicity through the AMPK/SIRT and Akt signaling axis mediated autophagy both *in vivo* and *in vitro*.

Although TP has been demonstrated to have anti-inflammatory, antitumor, antiarteriosclerosis, and analgesic effects, plenty of studies also report its reproductive toxicity both in human beings and animals. In line with the previous studies reported by Liu et al. [16], our results discovered that TP-induced reproductive toxicity in adult female rats are shown by the changes in pathology, the decrease of primary follicles and secondary follicles, and the increase of atretic follicles *in vivo*, as well as the dysregulation of some hormones both *in vivo* and *in vitro*. E2 is the most abundant and active estrogen in women. It is secreted by follicular granulosa cells and regulated by LH and FSH, which can promote the development of various organs of the reproductive system, facilitate endometrial hyperplasia and shedding, and maintain female secondary sexual characteristics [17]. AMH is secreted by the granular cells of small ovarian follicles, and its level is not affected by other exogenous hormone drugs or pregnancy. Thus, it is a reliable indicator for evaluating ovarian reserve and premature ovarian failure [18]. The level of INH B is tightly associated with the fertility potential that is negatively correlated with the FSH level and has a preferably predictive fertility potential compared with FSH [19]. Both LH and FSH are secreted by the basophils of the anterior pituitary. Therein, FSH can facilitate the proliferation and differentiation of granulosa cells of the follicle, promote the maturation of the follicles, and make the ovaries grow. LH and FSH play a synergistic effect to promote the discharge of mature eggs, so that the ruptured follicles form a corpus luteum to secrete estrogen and progesterone [20]. Therefore, the levels of E2, AMH, INH B, LH, and FSH can directly reflect the functional status of the ovaries. It has been demonstrated that the receptors of these hormones are widely expressed on the ovarian follicles [21, 22]. Moreover, these receptors may be the drug targets of both normal follicular development and ovarian diseases. For instance, Majdi Seghinsara et al. [23] show that the extracts of panax ginseng enhance the levels of FSH receptors and proliferation cell nuclear antigen (PCNA), which are involved in the improvement of follicular development. Besides, coenzyme Q10 upregulates the expression of FSH receptors and PCNA that ameliorates cyclophosphamide-induced premature ovarian failure [24]. Thus, XJCRTSZW might function on the receptors of these hormones to mitigate TP-induced reproductive toxicity in the present study. In addition, previous studies have shown that hypothalamus-pituitary-ovary axis (HPOA) mainly promoted follicle maturation and egg discharge through the feedback regulation mechanism of hormones and also had the role of regulating ovarian function, female menstrual cycle, and fertility function [25]. The abnormal function of HPOA is an important cause of female endocrine dysfunction and infertility [26]. However, XJCRTSZW treatment markedly reversed these abovementioned changes, which indicated that XJCRTSZW might also alleviate TP-induced reproductive toxicity through HPOA. Moreover, our previous study has reported that JiaJianCongRongTuSiZiWan (JJCRTSZW), of which the emperor medicines are the same as XJCRTSZW, improves blood circulation around the ovaries, ameliorates blood supply to the uterus, regulates the function of the HPOA, and corrects abnormal endocrine environment [27]. Taken together, we concluded that XJCRTSZW can alleviate TP-induced reproductive toxicity, although the specific mechanisms may need further studies in the future.

Mechanically, several responses are associated with TP-induced reproductive toxicity, including inflammation, oxidative stress, apoptosis, and autophagy [10]. In the present study, XJCRTSZW treatment notably reduced the TP-induced elevation of apoptosis rate, the levels of IL-1*β*, IL-6, and TNF-*α* both *in vivo* and *in vitro*, which suggested that XJCRTSZW can relieve TP-induced reproductive toxicity via apoptosis and inflammation. In addition, XJCRTSZW treatment also observably inverted the TP-induced alterations of SOD, GSH-Px, and MDA both *in vivo* and *in vitro*. SOD is an antioxidant enzyme that specifically scavenges oxygen-free radicals in the body that play an important role in the body's oxidation and antioxidant balance and can disproportionate superoxide anion-free radicals to produce hydrogen peroxide to be further converted into water by GSH-PX in the body [28]. MDA is a degradation product of lipid peroxides, reflecting the peroxidation degree of body fat [28]. Therefore, SOD and GSH-PX activity as well as MDA content reflect the degree of lipid peroxidation in the body and at the same time can indirectly reflect the severity of the body's cells attacked by free radicals and the ability to scavenge free radicals. Moreover, the supplementation of NAC further markedly reversed the TP-induced alterations of SOD, GSH-Px, and MDA *in vitro*. Since NAC has been demonstrated to have antioxidant properties in a growing number of studies [29, 30], we concluded that XJCRTSZW can ameliorate TP-induced reproductive toxicity via oxidative stress, in line with our previous study [31]. Furthermore, as the emperor medicines of XJCRTSZW, cistanche [32], cuscuta [33], and raspberry [34], as well as some minister medicines and adjuvants including rehmannia [35], Caulis Spatholobi [36], and eupatorium [37] have exhibited the antioxidant properties in a variety of diseases. Therefore, these results indicated that XJCRTSZW can ameliorate TP-induced reproductive toxicity via apoptosis, inflammation, and oxidative stress.

More and more evidence has been shown that TP is able to regulate the autophagic pathway in various cell lines and tissues, such as murine leukemia WEHI-3 cells and WEHI-3 generated leukemia mice [38], cardiomyocytes [39], neuronal cells [40], and cancer cells [41]. Moreover, TP can regulate autophagy via targeting multiple machineries or various signal pathways [42]. In the present study, we found XJCRTSZW treatment reduced the TP-induced enhancement of phosphorylated level of AMPK*α*-1 and SIRT1, but elevated the TP-induced decrease of phosphorylated level of Akt both *in vivo* and *in vitro*. Also, XJCRTSZW treatment antagonized the TP-induced changes of beclin-1, LC3-II/LC3-I, p62, and cleaved-caspase-3 both *in vivo* and *in vitro*. Emerging evidence indicates that both the AMPK/SIRT1 [43] and Akt [44] signaling axis are involved in autophagy, which can converge to mTOR [45, 46]. Therefore, XJCRTSZW treatment might inhibit autophagy via the AMPK/SIRT/mTOR and Akt/mTOR signaling axis. Consistently, the application of CQ, an autophagy inhibitor, further strengthened the effect of XJCRTSZW on the TP-induced changes abovementioned *in vitro*. Moreover, previous study has also reported that the AMPK/SIRT signaling pathway is associated with oxidative stress [47]. Oxidative stress is demonstrated to be a core contributor to TP-regulated autophagy [48]. Although our preprint results have demonstrated that the PINK1/Parkin signaling pathway-mediated mitophagy is strongly involved in the protective role of XJCRTSZW on oxidative stress injury in rats [31], its specific role in autophagy still needs more exploration. Thus, our results observed that the administration of NAC, an antioxidant, also further intensified the effect of XJCRTSZW on the TP-induced changes abovementioned *in vitro*. In brief, XJCRTSZW relieves TP-induced reproductive toxicity via the AMPK/SIRT and Akt signaling axis mediated autophagy.

In conclusion, XJCRTSZW can alleviate TP-induced reproductive toxicity via apoptosis, inflammation, and oxidative stress both *in vivo* and *in vitro*. Moreover, XJCRTSZW ameliorates TP-induced reproductive toxicity through AMPK/SIRT and Akt signaling axis mediated autophagy both *in vivo* and *in vitro*. However, our study also has some limitations. For instance, it has been reported that there is crosstalk between autophagy and apoptosis, as well as autophagy and oxidative stress regulated by TP [42]. Therefore, further research needs to explore the relationships between autophagy and apoptosis, as well as autophagy and oxidative stress after XJCRTSZW treatment. Moreover, TP has been revealed to regulate mitophagy [42]. Thus, the role of XJCRTSZW in TP-induced mitophagy, as well as the relationship between TP-induced autophagy and TP-induced mitophagy, needs deep study. Taken together, the results provide a theoretical basis for the clinical development of therapeutic drugs targeted to treat reproductive toxicity.

## Data Availability

The datasets used or analyzed during the current study are available from the corresponding author upon reasonable request.

## Supplementary Materials

Supplementary Materials The detection of oestrous cyclicity by papanicolaou stain and the analysis of active ingredients of XJCRTSZW by LC/MS.

## References

[1] Chen S. R., Dai Y., Zhao J., Lin L., Wang Y., Wang Y. (2018). A mechanistic overview of triptolide and celastrol, natural products from tripterygium wilfordii Hook F. *Frontiers in Pharmacology*.

[2] Song C., Wang Y., Cui L., Yan F., Shen S. (2019). Triptolide attenuates lipopolysaccharide-induced inflammatory responses in human endothelial cells: involvement of NF-*κ*B pathway. *BMC Complementary and Alternative Medicine*.

[3] Song W., Liu M., Wu J., Zhai H., Chen Y., Peng Z. (2019). Preclinical pharmacokinetics of triptolide: a potential antitumor drug. *Current Drug Metabolism*.

[4] Yuan K., Li X., Lu Q. (2019). Application and mechanisms of triptolide in the treatment of inflammatory diseases-a review. *Frontiers in Pharmacology*.

[5] Qian S. Z., Zhong C. Q., Xu Y. (1986). Effect of Tripterigium wilfordii Hook. f. on the fertility of rats. *Contraception*.

[6] Qian S. Z. (1987). A Chinese herb effective in male fertility regulation. *Contraception*.

[7] Hikim A. P., Lue Y. H., Wang C., Reutrakul V., Sangsuwan R., Swerdloff R. S. (2000). Posttesticular antifertility action of triptolide in the male rat: evidence for severe impairment of cauda epididymal sperm ultrastructure. *Journal of Andrology*.

[8] Gu J., Zhu C., Wang W., Wang L. (2001). The effects of Lei Gong Teng on reproductive hormones. *Journal of Traditional Chinese Medicine*.

[9] Chen X., Chen S. L. (2011). A woman with premature ovarian failure induced by Tripterygium wilfordii Hook.f. gives birth to a healthy child. *Fertility and Sterility*.

[10] Xi C., Peng S., Wu Z., Zhou Q., Zhou J. (2017). Toxicity of triptolide and the molecular mechanisms involved. *Biomedicine & Pharmacotherapy*.

[11] Mizushima N. (2007). Autophagy: process and function. *Genes & Development*.

[12] Krosch T. C., Sangwan V., Banerjee S. (2013). Triptolide-mediated cell death in neuroblastoma occurs by both apoptosis and autophagy pathways and results in inhibition of nuclear factor–kappa B activity. *The American Journal of Surgery*.

[13] Mujumdar N., Mackenzie T. N., Dudeja V. (2010). Triptolide induces cell death in pancreatic cancer cells by apoptotic and autophagic pathways. *Gastroenterology*.

[14] Li J., Su J., Guo Z. Q., Wu K. M. (2014). Wu keming’s experience in treating premature ovarian failure. *Hunan Journal of Traditional Chinese Medicine*.

[15] Ke X., Wang M., Wu K. M. (2015). Professor Wu Keming’s experience in the treatment of PCOS with integrated traditional Chinese and western medicine. *TCM clinical research*.

[16] Liu J., Jiang Z., Liu L. (2011). Triptolide induces adverse effect on reproductive parameters of female Sprague-Dawley rats. *Drug and Chemical Toxicology*.

[17] Nazari E., Suja F. (2016). Effects of 17*β*-estradiol (E2) on aqueous organisms and its treatment problem: a review. *Reviews on Environmental Health*.

[18] Suardi D., Permadi W., Djuwantono T., Hidayat Y. M., Bayuaji H., Edo Gautama G. P. (2021). Correlation of serum anti-mullerian hormone (AMH) level on ovarian volume in women with endometrioma. *International Journal of General Medicine*.

[19] de Gouveia Brazao C. A., Pierik F. H., Erenpreiss Y., de Jong F. H., Dohle G. R., Weber R. F. A. (2003). The effect of cryptorchidism on inhibin B in a subfertile population. *Clinical Endocrinology*.

[20] Palermo R. (2007). Differential actions of FSH and LH during folliculogenesis. *Reproductive BioMedicine Online*.

[21] Recchia K., Jorge A. S., Pessôa L. V. d. F. (2021). Actions and roles of FSH in germinative cells. *International Journal of Molecular Sciences*.

[22] Lee E. B., Chakravarthi V. P., Wolfe M. W., Rumi M. A. K. (2021). ER*β* regulation of gonadotropin responses during folliculogenesis. *International Journal of Molecular Sciences*.

[23] Majdi Seghinsara A., Shoorei H., Hassanzadeh Taheri M. M. (2019). Panax ginseng extract improves follicular development after mouse preantral follicle 3D culture. *Cell Journal*.

[24] Delkhosh A., Delashoub M., Tehrani A. A. (2019). Upregulation of FSHR and PCNA by administration of coenzyme Q10 on cyclophosphamide-induced premature ovarian failure in a mouse model. *Journal of Biochemical and Molecular Toxicology*.

[25] Wang F., Zhang Z. H., Xiao K. Z., Wang Z. C. (2017). Roles of hypothalamic-pituitary-adrenal axis and hypothalamus-pituitary-ovary axis in the abnormal endocrine functions in patients with polycystic ovary syndrome. *Zhongguo Yi Xue Ke Xue Yuan Xue Bao*.

[26] Mikhael S., Punjala-Patel A., Gavrilova-Jordan L. (2019). Hypothalamic-pituitary-ovarian axis disorders impacting female fertility. *Biomedicines*.

[27] Hu D. M., Wu K. M. (2010). Analysis of curative effect of JiaJianCongRongTuSiZiWan in the treatment of premature ovarian failur. *Gansu Traditional Chinese Medicine*.

[28] Askari M., Mozaffari H., Darooghegi Mofrad M. (2021). Effects of garlic supplementation on oxidative stress and antioxidative capacity biomarkers: a systematic review and meta-analysis of randomized controlled trials. *Phytotherapy Research*.

[29] Charron M. J., Williams L., Seki Y. (2020). Antioxidant effects of N-acetylcysteine prevent programmed metabolic disease in mice. *Diabetes*.

[30] Luan X., Yan Y., Zheng Q., Wang M., Chen W., Yu J. (2020). Excessive reactive oxygen species induce apoptosis via the APPL1-Nrf2/HO-1 antioxidant signalling pathway in trophoblasts with missed abortion. *Life Sciences*.

[31] Jin Y., Deng D., Liu M., Wu Y., Wu K. (2021). XinJiaCongRongTuSiZiWan protects tp-induced rats from oxidative stress injury via mitophagy mediated PINK1/parkin signaling pathway. https://www.researchsquare.com/article/rs-990305/v1.

[32] Mehanna E. T., El-Sayed N. M., Ibrahim A. K., Ahmed S. A., Abo-Elmatty D. M. (2018). Isolated compounds from Cuscuta pedicellata ameliorate oxidative stress and upregulate expression of some energy regulatory genes in high fat diet induced obesity in rats. *Biomedicine & Pharmacotherapy*.

[33] Hu Y., Huang J., Li Y. (2020). Cistanche deserticola polysaccharide induces melanogenesis in melanocytes and reduces oxidative stress via activating NRF2/HO-1 pathway. *Journal of Cellular and Molecular Medicine*.

[34] Garcia G., Nanni S., Figueira I. (2017). Bioaccessible (poly)phenol metabolites from raspberry protect neural cells from oxidative stress and attenuate microglia activation. *Food Chemistry*.

[35] Yang Y. H., Yang H., Li R. F. (2021). A Rehmannia glutinosa cinnamate 4-hydroxylase promotes phenolic accumulation and enhances tolerance to oxidative stress. *Plant Cell Reports*.

[36] Park H. R., Lee H., Lee J. J., Yim N. H., Gu M. J., Ma J. Y. (2018). Protective effects of Spatholobi Caulis extract on neuronal damage and focal ischemic stroke/reperfusion injury. *Molecular Neurobiology*.

[37] Melo A. S., Monteiro M. C., da Silva J. B. (2013). Antinociceptive, neurobehavioral and antioxidant effects of Eupatorium triplinerve Vahl on rats. *Journal of Ethnopharmacology*.

[38] Chan S. F., Chen Y. Y., Lin J. J. (2017). Triptolide induced cell death through apoptosis and autophagy in murine leukemia WEHI-3 cells in vitro and promoting immune responses in WEHI-3 generated leukemia mice in vivo. *Environmental Toxicology*.

[39] Zhou J., Xi C., Wang W., Yang Y., Qiu Y., Huang Z. (2015). Autophagy plays an important role in triptolide-induced apoptosis in cardiomyocytes. *Toxicology Letters*.

[40] Hu G., Gong X., Wang L. (2017). Triptolide promotes the clearance of *α*-synuclein by enhancing autophagy in neuronal cells. *Molecular Neurobiology*.

[41] Mujumdar N., Banerjee S., Chen Z. (2014). Triptolide activates unfolded protein response leading to chronic ER stress in pancreatic cancer cells. *American Journal of Physiology-Gastrointestinal and Liver Physiology*.

[42] Wei Y. M., Wang Y. H., Xue H. Q., Luan Z. H., Liu B. W., Ren J. H. (2019). Triptolide, a potential autophagy modulator. *Chinese Journal of Integrative Medicine*.

[43] Heshmati M., Soltani A., Sanaei M. J. (2020). Ghrelin induces autophagy and CXCR4 expression via the SIRT1/AMPK axis in lymphoblastic leukemia cell lines. *Cellular Signalling*.

[44] Hou G., Jia A., Yang L. (2020). OP16 induces deadly autophagy and apoptosis of cells by inhibiting Akt in esophageal squamous cell carcinoma. *Molecular and Cellular Biochemistry*.

[45] Ganesan R., Hos N. J., Gutierrez S. (2017). Salmonella Typhimurium disrupts Sirt1/AMPK checkpoint control of mTOR to impair autophagy. *PLoS Pathogens*.

[46] Huang M. L., Qi C. L., Zou Y. (2020). Plac8-mediated autophagy regulates nasopharyngeal carcinoma cell function via AKT/mTOR pathway. *Journal of Cellular and Molecular Medicine*.

[47] Ren H., Shao Y., Wu C., Ma X., Lv C., Wang Q. (2020). Metformin alleviates oxidative stress and enhances autophagy in diabetic kidney disease via AMPK/SIRT1-FoxO1 pathway. *Molecular and Cellular Endocrinology*.

[48] Wang J., Jiang Z., Ji J. (2013). Gene expression profiling and pathway analysis of hepatotoxicity induced by triptolide in Wistar rats. *Food and Chemical Toxicology*.

