# Supplementary material for: Protective Effect of XinJiaCongRongTuSiZiWan on the Reproductive Toxicity of Female Rats Induced by Triptolide
Source: Evid Based Complement Alternat Med. 2022 Jun 6;2022:3642349. doi: 10.1155/2022/3642349 (PMC9192320; doi:10.1155/2022/3642349)
Supplement: Supplementary Materials — The detection of oestrous cyclicity by papanicolaou stain and the analysis of active ingredients of XJCRTSZW by LC/MS. [file 3642349.f1.doc]

**Protective effect of XinJiaCongRongTuSiZiWan on the** **reproductive toxicity of female rats induced by** **triptolide**

1 Materials and methods

1.1 Papanicolaou stain

32 health rats were acclimated to standard laboratory conditions for 7 days, and then the vaginal exfoliated cells of rats were smeared on glass slides every 9:00 am for 10 consecutive days. The cell samples on the slides were subsequently subjected to papanicolaou stain. In brief, smears were first fixed with 95% ethyl alcohol for 15 min, and then were stained with hematoxylin for 2 min. After washed with running water two times, samples were further stained with a mix of EA-36/Orange G-6 for 2 min. Next, smears were treated with xylene transparent, mounted coverslip, and observed under a microscope.

1.2 Liquid chromatography-mass spectrometry (LC/MS) analysis analysis

Before LC/MS analysis, 2.5g XJCRTSZW with 10mL hot water was subjected with ultrasonic for 10min. Then, samples were centrifuged for 10min at 12000 r/min to obtain the supernatants. After the supernatants were filtered with 0.45 µm membrane, 10 µL samples were prepared for further test. The High-Performance Liquid Chromatography (HPLC) system LC-30 (SHIMADZU, Kyoto, Japan) was connected with a mass spectrometer SCIEX 5600 (AB Sciex Instruments, United States). The chromatographic separation was carried out with a SHIMADZU InerSustain C18 (100×2.1 mm,2 µm). The column temperature was set at 35°C. Mobile phase A was acetonitrile; mobile phase B was 0.1% HCOOH-H_2_O. The chromatographic conditions were listed as follows: 5% A and 95% B at 0 and 2 min; 20% A and 80% B at 4 min; 15% A and 75% B at 12 min; 46% A and 54% B at 14 min; 100% A and 0% B at 26 and 28 min; 5% A and 95% B at 29 and 30 min, with an injection volume 10 μL. Modes with electrospray ionization (ESI) positive ion and negative ion were used in the present study respectively. The ESI source conditions were as follows: Ion Source Gas1 (Gas 1): 50; Gas 2: 50; Curtain Gas (CUR): 25; Source Tempreture: 500℃ (positive ion) and 450℃ (negative ion); Ion Sapary Voltage Floating (ISVF) 5500 V (positive ion) and 4400 V (negative ion); TOF MS scan range: 100-1200Da; product ion scan range: 50-1000 Da; TOF MS scan accumulation time 0.2s; and product ion scan accumulation time 0.01s. The secondary mass spectrum was acquired by information dependent acquisition (IDA), and adopts high sensitivity mode: Declustering potential (DP): ±60 V, and Collision Energy: 35±15 eV.

2 Results

2.1 The detection of oestrous cyclicity

To pick the rats with normal oestrous cyclicity, the vaginal exfoliated cells of rats were smeared on glass slides and administrated with papanicolaou stain. Four oestrous stages, including proestrus (P), estrus (E), metaestrus (M) and anestrus (A) were distinguished according to the papanicolaou stain and used to estimate the oestrous cyclicity. The rats with a oestrous cyclicity with approximately 5 days were selected for subsequent experiments. Then, some of these rats were modeled with 400 μg/kg.d TP for continuous 40 days to induce the reproductive toxicity. The TP-induced rats showed the oestrous cyclicity extended to 7 days or even more, which suggested that the estrous cycle was disordered. The representative results were listed at Figure S1.


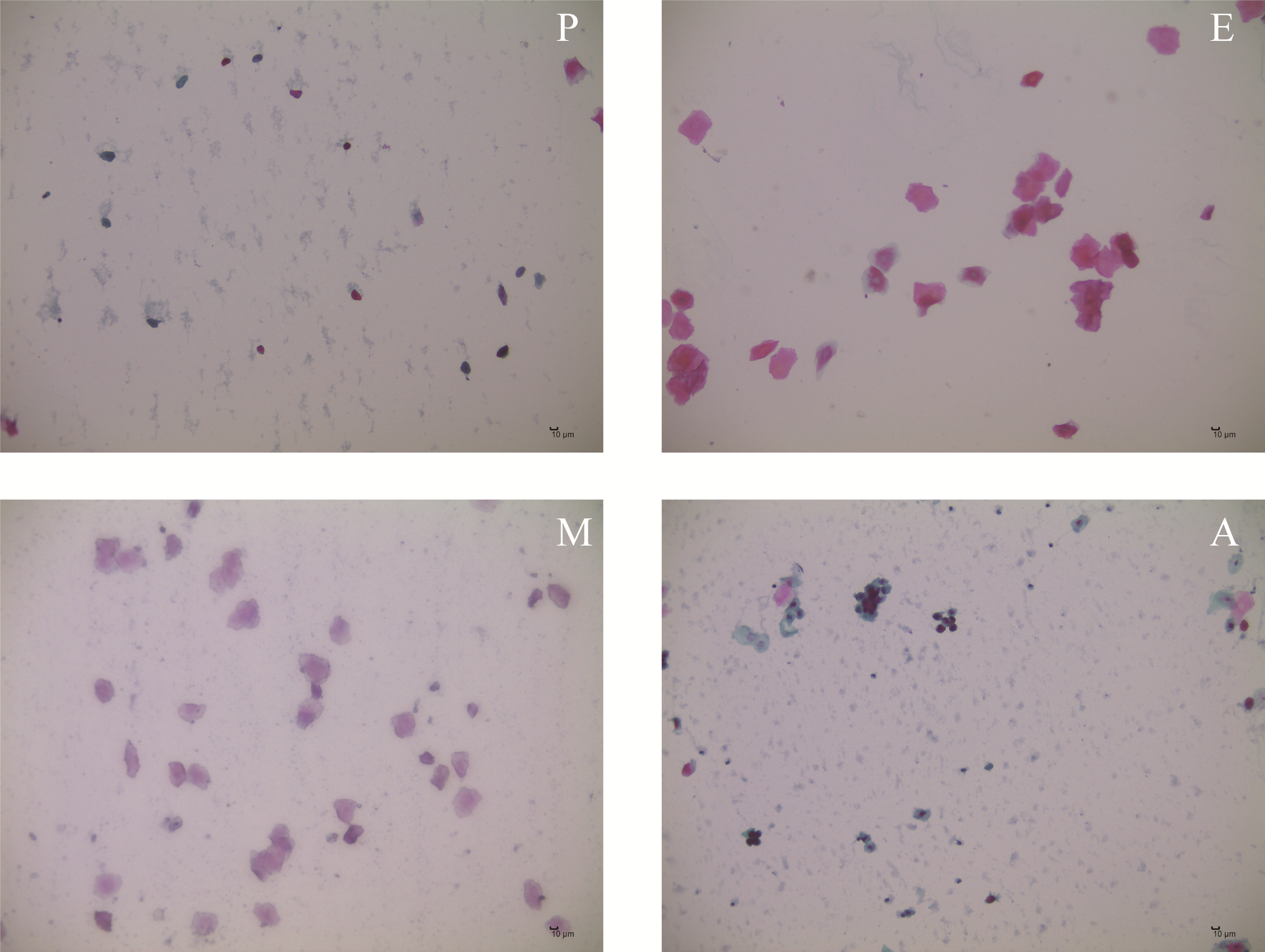


Fig S1 Morphological changes of vaginal exfoliated cells in each stage of the estrous cycle in rats. Four oestrous stages, including proestrus (P), estrus (E), metaestrus (M) and anestrus (A) were exhibited in the rats. 200×.

2.2 The analysis of active ingredients of XJCRTSZW

The chromatograms of positive and negative ion of XJCRTSZW sample were shown in Fig S2. The raw data of LC-MS were imported into MS-DIAL 4.60 software [1] for preprocessing, including peak extraction, noise removal, deconvolution, peak alignment. The extracted peak information was compared with MassBank, Respect, and GNPS databases. The detailed active ingredients of XJCRTSZW were listed in Table S1.


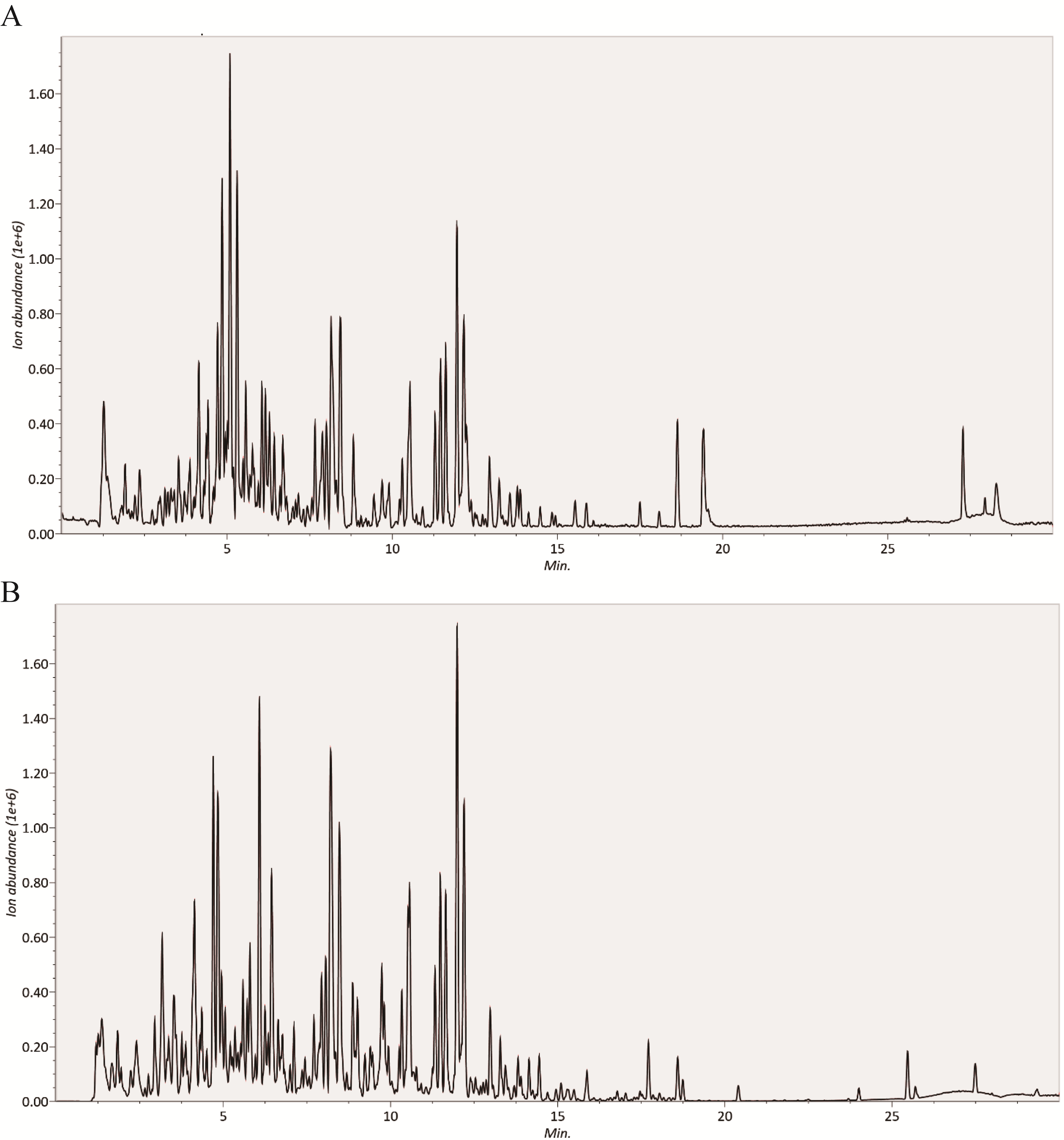


Fig S2 The chromatograms of positive and negative ion of XJCRTSZW sample. (A) The chromatograms of positive ion of XJCRTSZW sample. (B) The chromatograms of negative ion of XJCRTSZW sample.

**References**

1. Tsugawa, H., et al.*, MS-DIAL: data-independent MS/MS deconvolution for comprehensive metabolome analysi*s. Nat Methods, 2015**.** 12(6): p. 523-6.
